# Indication-specific adverse event profiles of semaglutide and liraglutide: a FAERS disproportionality analysis

**DOI:** 10.3389/fphar.2026.1936967

**Published:** 2026-09-29

**Authors:** Samuel A. Fisch, Eitan J. Neugut, Nicole Cornet, Daniela Quigee, Alfred I. Neugut, Daniel E. Freedberg

**Affiliations:** 1 Department of Epidemiology, Mailman School of Public Health, Columbia University, New York, NY, United States; 2 Department of Statistics, Columbia University, New York, NY, United States; 3 Department of Medicine, Vagelos College of Physicians & Surgeons, Columbia University, New York, NY, United States

**Keywords:** disproportionality analysis, FDA Adverse Event Reporting System, glucagon-like peptide-1, glucagon-like peptide-1 receptor agonists, liraglutide, semaglutide

## Abstract

**Objective:**

By 2021, regulatory agencies had approved two glucagon-like peptide-1 receptor agonists (GLP-1RAs), semaglutide and liraglutide, for the treatment of type 2 diabetes and obesity. Since then, concerns have emerged about whether adverse events (AEs) differ by GLP-1RA or indication.

**Research design and methods:**

We analyzed the Food and Drug Administration Adverse Event Reporting System (FAERS) reports (2021–2025) to compare adverse events associated with semaglutide and liraglutide indications. Comparisons with metformin and orlistat helped distinguish drug-related signals from disease-related signals. We defined safety signals by a positive reporting odds ratio and at least one other positive disproportionality metric.

**Results:**

Among 8,213,105 FAERS reports, 33,259 met the inclusion criteria. Semaglutide generated more reports for type 2 diabetes than for obesity (13,168 vs. 5,444), and liraglutide showed a similar pattern (1,201 vs. 837). GLP-1RAs exhibited gastrointestinal AEs. Semaglutide showed signals for slowed gastrointestinal motility across both indications, particularly impaired gastric emptying, in both type 2 diabetes and obesity reports (ROR = 8.35, 95% CI = 1.16–59.97 and ROR = 4.01, 95% CI = 2.12–7.58, respectively). Liraglutide demonstrated signals for more severe gastrointestinal AEs, including abdominal distention in type 2 diabetes (ROR = 1.46, 95% CI = 1.12–1.91) and pancreatitis in obesity (ROR = 2.90, 95% CI = 1.91–4.39) reports. Additional notable safety signals included optic ischemic neuropathy with semaglutide (ROR = 4.42, 95% CI = 1.41–13.87) and psychiatric AEs, including suicidal ideation (ROR = 5.98, 95% CI = 1.24–24.19).

**Conclusion:**

We identified robust signals for GLP-1RAs, including impaired gastric emptying, ileus, and ischemic optic neuropathy. Additionally, we identified signals for suicidal ideation. Future studies are needed to evaluate for causal relationships.

## Highlights

- Why did we undertake this study? In recent years, GLP-1RA usage has increased dramatically among patients with obesity. The consensus is that GLP-1RAs cause gastrointestinal adverse effects, but their full safety profile remains unknown.
- What is the specific question(s) we wanted to answer? Our goal was to compare adverse events among (1) GLP-1RAs for diabetes vs. obesity; (2) semaglutide vs. liraglutide; and (3) GLP-1RAs vs. traditional diabetes and obesity drugs.
- What did we find? We identified novel signals for eye, metabolic, and psychiatric adverse events while confirming established adverse effects related to impaired gastric emptying.
- What are the implications of our findings? The novel AEs of optic ischemic neuropathy and suicidal ideation warrant further investigation, especially regarding semaglutide.

## Introduction

Over the past decade, glucagon-like peptide-1 receptor agonist (GLP-1RA) drugs have emerged as leading therapeutic agents for both diabetes and obesity, with approximately 1 in 8 adults reporting usage (Harris, 2024; Moiz et al., 2025). Although the U.S. Food and Drug Administration (FDA) has approved several GLP-1RAs for either type 2 diabetes or weight loss (hereafter termed obesity), it has approved only liraglutide and semaglutide for both indications. Tirzepatide, a dual GIP/GLP-1 receptor agonist, is also FDA-approved for both indications (Syed, 2022).

The FDA approved liraglutide for type 2 diabetes in 2010 (Center for Drug Evaluation and Research, 2010) and for obesity in 2014 (U.S. Food and Drug Administration, 2014); semaglutide, a newer, longer-acting GLP-1RA, received FDA approval for type 2 diabetes in 2017 (U.S. Food and Drug Administration, 2017) and for obesity in 2021 (U.S. Food and Drug Administration, 2021).

Although patients have experienced substantial benefits from GLP-1RAs, the rapid pace of public adoption has raised concerns that safety monitoring may not be sufficient to track potential risks in real time. Post-marketing surveillance reports have documented gastrointestinal adverse events (AEs) (Sodhi et al., 2023), acute pancreatitis (Singh et al., 2013), gallbladder disease (Ramírez-Mejía et al., 2025), acute kidney injury (Dong and Sun, 2022), ophthalmic complications (Ramsey et al., 2025), thyroid cancer (Bezin et al., 2023), and rare reports of suicidal ideation (Tang et al., 2024). Prior retrospective cohort studies have consistently found that GLP-1RAs are associated with AEs related to slowing of the gastrointestinal tract (Sodhi et al., 2023), but these studies have limited ability to evaluate rare AEs or identify novel AEs.

Post-marketing surveillance reports can generate signals for novel AEs, but interpreting these data can be challenging as patients with type 2 diabetes and obesity are inherently at increased risk for many previously reported AEs. Despite their limitations, signal reports remain valuable tools for hypothesis generation. This is the first study to evaluate AEs associated with semaglutide and liraglutide within their approved indications of type 2 diabetes and obesity using data from the Food and Drug Administration Adverse Event Reporting System (FAERS). We assessed for signals when comparing semaglutide with liraglutide and for signals that were GLP1-class-specific, by comparing both drugs against their nearest comparators, metformin for type 2 diabetes and orlistat for obesity.

## Materials and methods

### Data source

The FAERS database serves as one of the largest global repositories for documenting drug AE reports in real-world settings. Healthcare professionals, manufacturers, and patients submit spontaneous AE reports to FAERS. The FDA publishes quarterly releases that include seven files: patient demographics, drug/biologic information, reactions, patient outcomes, reporting source, drug start and end dates, and indications (Potter et al., 2025).

Our study restricted our analysis to reports from 2021 to the third quarter of 2025. We selected 2021 as the start date to ensure comparable prescribing periods for both medications (Ukhanova et al., 2025).

### Data acquisition and processing

We matched all FAERS files and their terminology with the Medical Dictionary for Regulatory Activities (MedDRA) vocabulary, applying standardized concepts for AE, including preferred terms (PTs) and system organ classes (SOCs). The MedDRA hierarchy is organized into five levels. SOCs represent the broadest and highest level of the hierarchy, whereas PTs are distinct medical descriptors located at the second-lowest level. The most granular lower-level terms (LLTs) are not available within the publicly available version of FAERS (Potter et al., 2025). For example, the PT “optic ischemic neuropathy” includes all clinically recognized forms, such as anterior ischemic optic neuropathy (AION), nonarthritic anterior ischemic optic neuropathy (NAION), and other related disorders including LLTs; however, we are unable to distinguish between these clinical categories, all of which fall under the PT of optic ischemic neuropathy.

We also standardized indication terms for type 2 diabetes and obesity to ensure consistency across reports. After harmonization, we removed duplicate reports following FDA-recommended deduplication procedures (Potter et al., 2025). We deemed 13 of the 29 SOCs to be clinically relevant and excluded the remaining SOCs from the analysis.

We restricted our dataset to include only case reports that (1) specified the ingredient name of the target drug, (2) designated the drug as the primary suspect (PS), and (3) reported a serious AE. We characterized reports as type 2 diabetes or obesity based on the listed indication codes. We excluded patients with both indications.

From the eligible reports, we extracted baseline characteristics including sex, age, reporting source, geographic region, and reporting year. Additionally, we extracted patient outcomes including whether AEs resulted in death or were life-threatening. In FAERS, the original reporter of the event assigns outcome seriousness categories and death, and these outcome categories are neither validated nor reassigned by the FDA. We did not adjust for dose, treatment duration, comorbidities, or concomitant medications because, although some of these variables are included in the FAERS, they are often incomplete (Potter et al., 2025).

### Data algorithms

We employed four data-mining algorithms commonly used in pharmacovigilance databases to detect and quantify AE signals (Almenoff et al., 2005; Sakaeda et al., 2013). These four algorithms—reporting odds ratio (ROR), proportional reporting ratio (PRR), information component (IC), and empirical Bayesian geometric mean (EBGM)—are based on different statistical frameworks, including both frequentist (non-Bayesian) and Bayesian approaches, serving complementary pharmacovigilance objectives (van Puijenbroek et al., 2002; Hauben et al., 2005; Bate and Evans, 2009).

In brief, the ROR compares the odds of a specific adverse event for a given drug with those for all other drugs. We calculated the ROR and its 95% confidence interval (CI) as the ratio of the odds of a specific PT occurring with the target drug versus all other drugs pertinent to our research question (i.e., semaglutide, liraglutide, metformin, and orlistat). We defined a signal as ROR lower than 95% CI > 1.0 and ≥3 cases.

The PRR compares the proportion of reports for a specific adverse event associated with a drug to the corresponding proportion for all other drugs. We defined a signal as PRR ≥2, chi-squared ≥4, and ≥3 cases.

The IC quantifies the deviation between observed and expected reporting of a drug–adverse event pair, with positive values indicating higher-than-expected reporting. We calculated the IC as the base-2 logarithm of the ratio of observed-to-expected reporting frequency. We defined a signal as IC025, the lower bound of the 95% credibility interval, >0.

The EBGM estimates the strength of drug–adverse event associations by shrinking observed reporting ratios toward the overall mean, thereby reducing the influence of random variation and false-positive signals. We defined a signal as an EB05, the lower bound of the 90% one-sided credibility interval, >2.

We included a signal in our study when it registered a positive ROR (lower limit of 95% CI > 1.0, N ≥ 3) and satisfied at least one other algorithm (PRR, IC, or EBGM). We prioritized the ROR to ensure adequate sensitivity for initial signal identification and ease of interpretation, while subsequent algorithms provided complementary validation and robustness.

### Statistical analysis

Our analysis included four primary comparisons: (1) semaglutide versus liraglutide restricted to the type 2 diabetes indication, (2) semaglutide versus liraglutide restricted to the obesity indication, and the corresponding inverse comparisons for each indication. We also conducted two secondary comparisons: (1) GLP-1RAs as a class (semaglutide and liraglutide) versus metformin for the type 2 diabetes indication and (2) GLP-1RAs versus orlistat for the obesity indication. Within each comparison, we performed two analyses: one at the SOC level to identify broad categories of AEs that differed between groups, and another at the PT level to identify specific AEs driving these differences.

Among the predefined clinically relevant SOCs, we assessed the disproportionality of reports using the ROR. We assessed PTs as previously discussed, requiring a positive ROR (lower limit of 95% CI > 1.0, N ≥ 3) and at least one additional positive algorithm (PRR, IC, or EBGM). For both SOCs and PTs, we constructed side-by-side forest plots.

We conducted all statistical analyses using R (version 4.5.1) and used the *faers* package to process FAERS data and perform disproportionality analyses (R Core Team, 2025; Peng et al., 2026).

## Results

### AE reports

From 2021 through the third quarter of 2025, we captured 8,213,105 AE reports. Of these, we identified 84,559 primary PS drug reports involving semaglutide, liraglutide, metformin, and orlistat. A total of 40,441 reports remained after filtering by indication, and 33,259 remained after filtering out SOCs deemed not clinically relevant. Semaglutide generated more type 2 diabetes-related reports than obesity-related reports (13,168 vs. 5,444), and liraglutide showed a similar pattern (1,201 vs. 837). Among the comparators, metformin accounted for 12,206 type 2 diabetes reports, and orlistat accounted for 53 obesity reports.

### Characteristics of AE reports

Table 1 summarizes the clinical characteristics of the AE reports. Metformin users had the highest median age among type 2 diabetes-indicated treatments at 67 years (IQR 58–76), followed by semaglutide at 65 years (56–72) and liraglutide at 64 years (54–71). Metformin reports also included the largest proportion of individuals aged ≥65 years (57.4%), compared with semaglutide (51.2%) and liraglutide (46.8%). Among obesity-indicated treatments, orlistat and semaglutide users had the highest median age at 53 years (40–65) and 53 years (42–63), respectively, followed by liraglutide at 49 years (39–58). Individuals aged 45–64 years accounted for the largest proportion of reports across all obesity treatments. Orlistat reports showed the highest proportion of female individuals among obesity-indicated treatments (87.2%), followed by liraglutide (84.7%) and semaglutide (78.9%). In type 2 diabetes-indicated reports, semaglutide showed the highest proportion of female individuals (61.5%), resulting in a more balanced sex distribution overall.

**TABLE 1:** Report characteristics across drugs and indications.

Characteristic	Type 2 diabetes			Obesity		
	Semaglutide	Liraglutide	Metformin	Semaglutide	Liraglutide	Orlistat
N (reports)	13,168	1,201	12,206	5,444	837	53
Age						
Median age, years (IQR)	65 (56–72)	64 (54–71)	67 (58–75)	53 (42–63)	49 (39–58)	53 (40–65)
Age 18–44, n (%)	749 (8.2)	92 (10.0)	673 (6.7)	1,141 (30.9)	247 (40.6)	13 (32.5)
Age 45–64, n (%)	3,695 (40.6)	396 (43.2)	3,585 (35.9)	1,717 (46.5)	283 (46.5)	16 (40.0)
Age ≥65, n (%)	4,660 (51.2)	429 (46.8)	5,739 (57.4)	833 (22.6)	79 (13.0)	11 (27.5)
Age missing^†^, n (%)	4,064 (30.9)	284 (23.7)	2,209 (18.1)	1,753 (32.2)	228 (27.2)	13 (24.5)
Sex						
Female, n (%)	7,585 (61.5)	676 (57.5)	5,646 (51.6)	4,059 (78.9)	683 (84.7)	41 (87.2)
Male, n (%)	4,756 (38.5)	500 (42.5)	5,302 (48.4)	1,087 (21.1)	123 (15.3)	6 (12.8)
Unknown sex^†^, n (%)	827 (6.3)	25 (2.1)	1,258 (10.3)	298 (5.5)	31 (3.7)	6 (11.3)
Geographic origin						
US, n (%)	10,783 (82.0)	603 (50.3)	2,639 (21.8)	4,032 (74.1)	310 (37.0)	42 (84.0)
EU, n (%)	755 (5.7)	71 (5.9)	5,159 (42.3)	390 (7.2)	120 (14.3)	1 (1.9)
Other region, n (%)	1,617 (12.3)	526 (43.8)	4,294 (35.2)	1,021 (18.8)	407 (48.6)	7 (13.2)
Report outcomes						
Outcome records	9,746	1,108	18,323	3,785	820	16
Death, n (%)	181 (1.9)	31 (2.8)	1,126 (6.2)	61 (1.6)	13 (1.6)	0 (0.0)
Hospitalization, n (%)	3,365 (34.5)	429 (38.7)	7,122 (38.9)	1,121 (29.6)	250 (30.5)	4 (25.0)
Disability, n (%)	355 (3.6)	24 (2.2)	276 (1.5)	143 (3.8)	28 (3.4)	1 (6.3)
Life-threatening, n (%)	340 (3.5)	35 (3.2)	2,401 (13.1)	166 (4.4)	38 (4.6)	0 (0.0)
Reporter type						
Reporter records	792	59	227	317	32	3
Health professional, n (%)	276 (34.8)	45 (76.3)	130 (57.3)	101 (31.9)	10 (31.2)	2 (66.7)
Consumer, n (%)	501 (63.3)	13 (22.0)	94 (41.4)	209 (65.9)	21 (65.6)	1 (33.3)
Lawyer, n (%)	0 (0.0)	0 (0.0)	0 (0.0)	0 (0.0)	0 (0.0)	0 (0.0)
Other/Unknown, n (%)	15 (1.9)	1 (1.7)	3 (1.3)	7 (2.2)	1 (3.1)	0 (0.0)

^†^ Age-category percentages are calculated among reports with non-missing age; age missing is calculated using all reports as the denominator. Sex percentages are calculated among reports with known sex; unknown sex is calculated using all reports as the denominator.

Metformin and liraglutide accounted for the highest hospitalization rates among type 2 diabetes-indicated reports (38.9% and 38.7%, respectively), followed by semaglutide (34.5%). In contrast, obesity-indicated reports for all drugs showed hospitalization rates below 30.5%. Metformin demonstrated the highest reporting rate of life-threatening reactions at 13.1%, while semaglutide and liraglutide showed comparable rates, approximately 3.4%. In the type 2 diabetes cohort, metformin accounted for the highest proportion of deaths (6.2%), followed by liraglutide (2.8%) and semaglutide (1.9%). In the obesity cohort, liraglutide and semaglutide had identical proportions of reported deaths (1.6%).

### Safety signal classes for semaglutide vs. liraglutide

Among type 2 diabetes-indicated reports, semaglutide demonstrated significantly greater reporting than liraglutide for gastrointestinal disorders (ROR = 1.67, 95% CI = 1.48–1.88; N = 7,633; Figure 1) and metabolism and nutrition disorders (ROR = 1.33, 95% CI = 1.16–1.54; N = 3,596). Broadly, the obesity-indicated reports were directionally similar to the diabetes reports; however, only metabolism and nutrition disorders reached statistical significance (ROR = 1.40, 95% CI = 1.18–1.67; N = 1,533).

**Figure 1:**
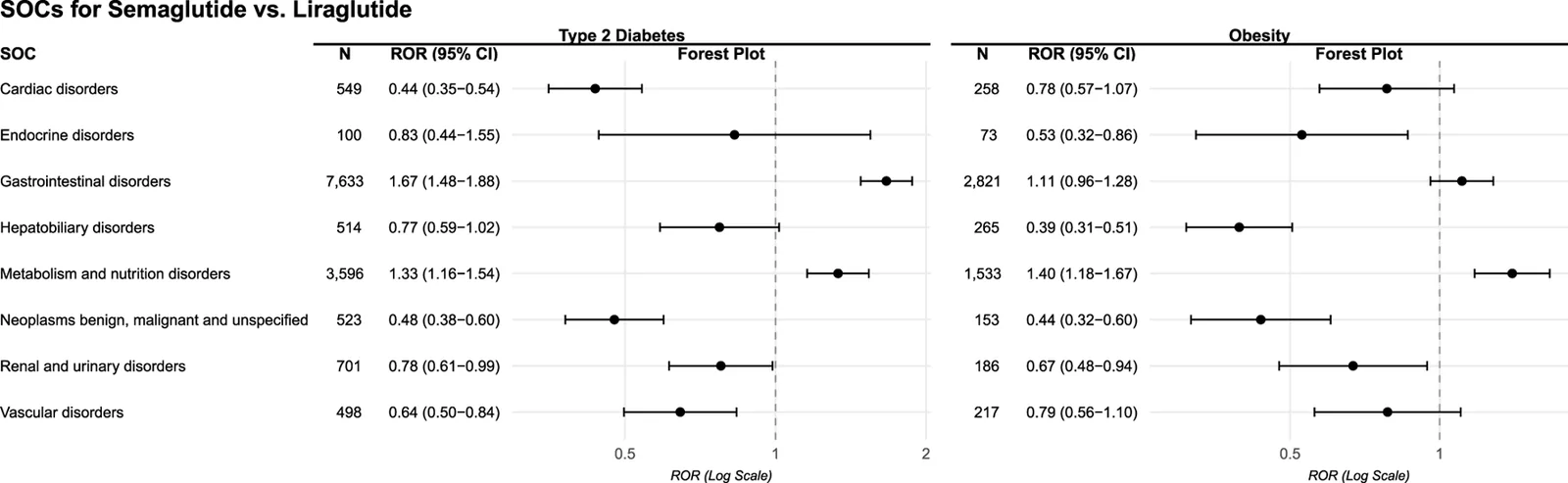
Reports of AEs for each system organ class by GLP‐1RA type and indication. *ROR, reported odds ratio. Note: The inverse of this figure shows the reports of AEs for each system organ class by GLP‐1RA type and indication for liraglutide vs. semaglutide.

### Safety signal classes for liraglutide vs. semaglutide

Among type 2 diabetes-indicated reports, liraglutide demonstrated significantly greater reporting than semaglutide for cardiac disorders (ROR = 2.27, 95% CI = 1.85–2.86; N = 109), benign, malignant, and unspecified neoplasms (ROR = 2.10, 95% CI = 1.68–2.63; N = 96), renal and urinary disorders (ROR = 1.29, 95% CI = 1.01–1.63; N = 81), and vascular disorders (ROR = 1.55, 95% CI = 1.20–2.01; N = 69). Similarly, among obesity-indicated reports, liraglutide demonstrated significantly greater reporting than semaglutide for endocrine disorders (ROR = 1.89, 95% CI = 1.16–3.09; N = 21), hepatobiliary disorders (ROR = 2.53, 95% CI = 1.98–3.24; N = 96), benign, malignant, and unspecified neoplasms (ROR = 2.29, 95% CI = 1.66–3.17; N = 52), and renal and urinary disorders (ROR 1.49, 95% CI 1.06–2.10; N = 42).

### GLP-1RAs as a class vs. metformin

We pooled semaglutide and liraglutide and compared them as a class against metformin within type 2 diabetes-indicated reports. We observed significant disproportionality signals for eye disorders (ROR = 2.54, 95% CI = 2.29–2.81; N = 1,521; gastrointestinal disorders (ROR = 4.17, 95% CI = 3.96–4.40; N = 8,177), benign, malignant, and unspecified neoplasms (ROR = 1.38, 95% CI = 1.21–1.57; N = 619), nervous system disorders (ROR = 1.11, 95% CI = 1.05–1.18; N = 3,197), and psychiatric disorders (ROR = 1.43, 95% CI = 1.31–1.56; N = 1,460).

### GLP-1RAs as a class vs. orlistat

We pooled semaglutide and liraglutide and compared them as a class against orlistat within obesity-indicated reports. We observed significant disproportionality signals for metabolism and nutrition disorders (ROR = 19.55, 95% CI = 2.70–141.50; N = 1,716) and psychiatric disorders (ROR = 7.57, 95% CI = 1.04–54.82; N = 798). Owing to the limited number of orlistat reports (n = 53), we could not estimate several SOC-level RORs.

### AEs for semaglutide vs. liraglutide in type 2 diabetes reports

We compared safety signal PTs between semaglutide and liraglutide in the type 2 diabetes-indicated reports (Figure 2). Semaglutide demonstrated stronger disproportionality signals for gastrointestinal dysmotility than liraglutide. The strongest signals included impaired gastric emptying (ROR = 6.10, 95% CI = 3.81–9.75; N = 1,118), constipation (ROR = 3.66, 95% CI = 2.65–5.06; N = 1,441), ileus (ROR = 2.73, 95% CI = 1.35–5.54; N = 237), and intestinal obstruction (ROR = 2.68, 95% CI = 1.57–4.58; N = 403). Semaglutide additionally showed increased reporting of food craving (ROR = 5.42, 95% CI = 1.34–21.96; N = 118), increased appetite (ROR = 2.57, 95% CI = 1.36–4.84; N = 278), and dehydration (ROR = 2.51, 95% CI = 1.67–3.79; N = 642). Semaglutide demonstrated stronger disproportionality signals for neuropsychiatric and sensory AEs. Prominent signals included dysgeusia (ROR = 8.35, 95% CI = 1.16–59.97; N = 91), suicidal ideation (ROR = 5.98, 95% CI = 1.48–24.19; N = 130), and depression (ROR = 2.27, 95% CI = 1.20–4.28; N = 246). We also identified ophthalmologic adverse events, including optic ischemic neuropathy (ROR = 4.42, 95% CI = 1.41–13.87; N = 144).

**Figure 2:**
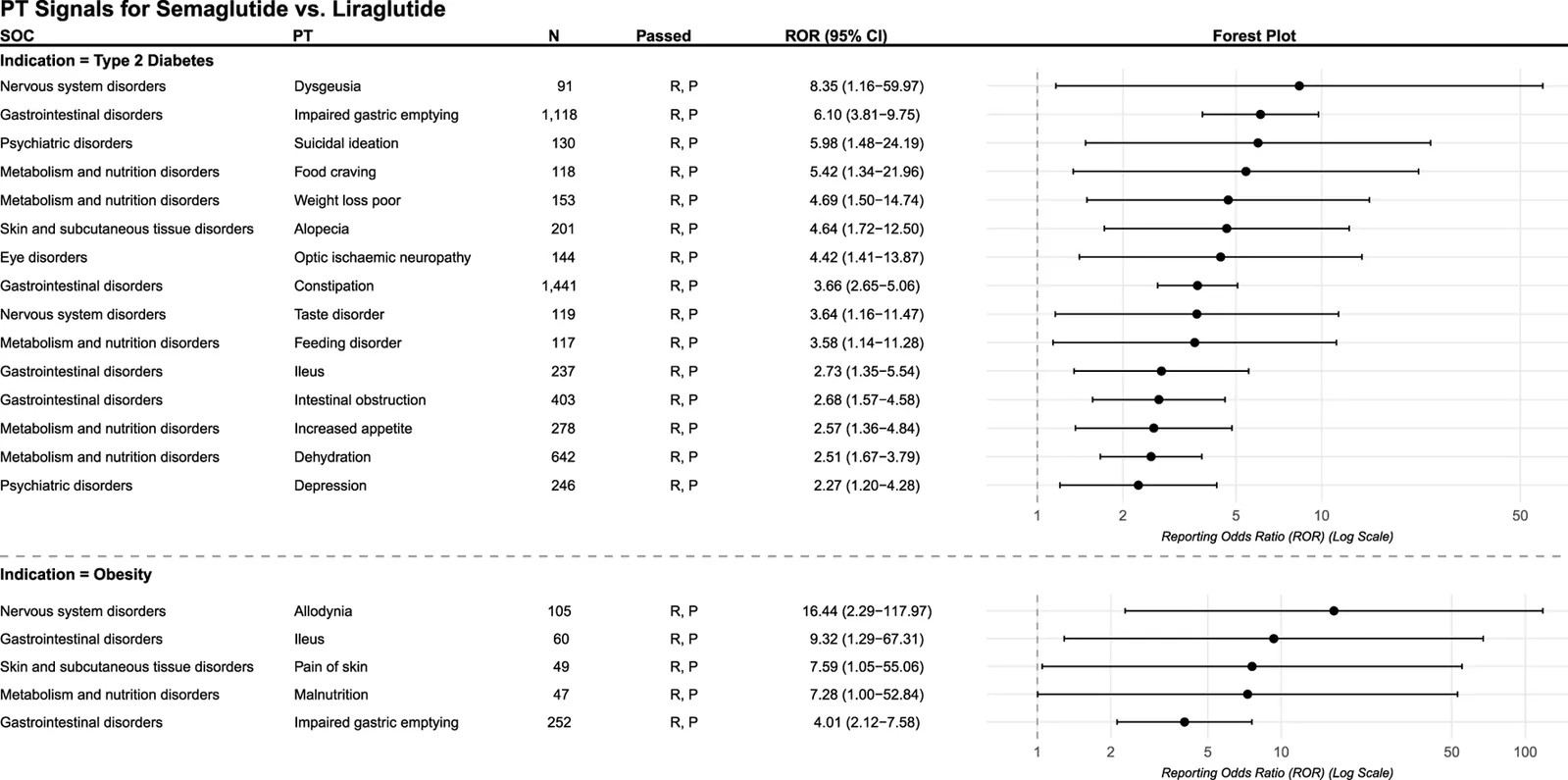
Comparative safety signals between semaglutide and liraglutide by indication. *R, reported odds ratio; P, proportional reporting ratio; I, information component; E, empirical Bayes geometric mean.

In contrast, liraglutide demonstrated stronger disproportionality signals for cardiac and hepatobiliary adverse events than semaglutide (Figure 3). Cardiac adverse event signals included myocardial ischemia (ROR = 14.66, 95% CI = 3.28–65.60; N = 4), and cardio-respiratory arrest (ROR = 6.59, 95% CI = 1.57–27.62; N = 86). Hepatobiliary adverse events included drug-induced liver injury (ROR = 7.09, 95% CI = 3.06–16.42; N = 9) and cholangitis (ROR = 3.67, 95% CI = 1.45–9.26; N = 6).

**Figure 3:**
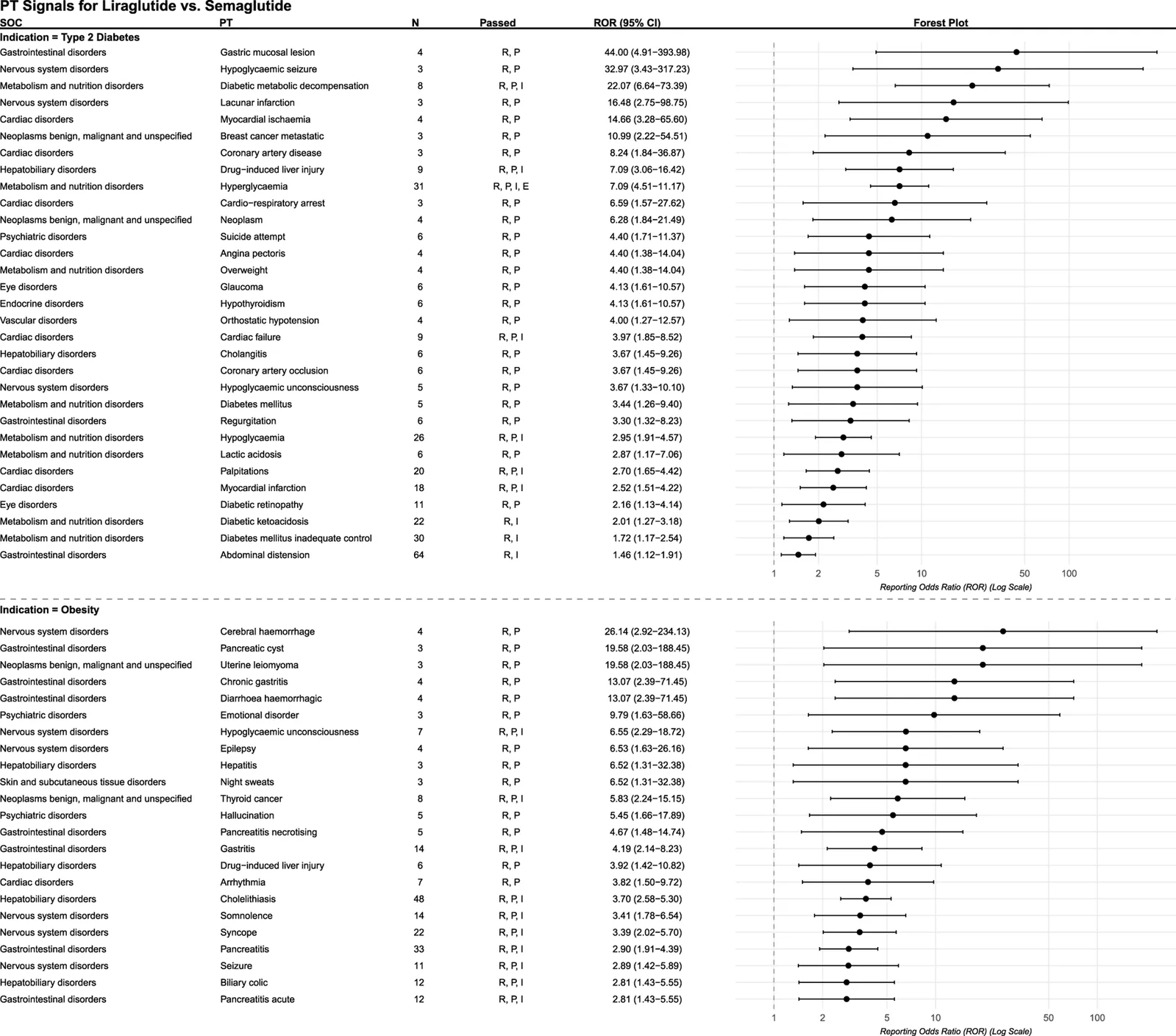
Comparative safety signals between liraglutide and semaglutide by indication. *R, reported odds ratio; P, proportional reporting ratio; I, information component; E, empirical Bayes geometric mean.

### AEs for semaglutide vs. liraglutide in obesity reports

Within the obesity indication cohort, semaglutide similarly demonstrated stronger signals for gastrointestinal dysmotility-related AE, including ileus (ROR = 9.32, 95% CI = 1.29–67.31; N = 60) and impaired gastric emptying (ROR = 4.01, 95% CI = 2.12–7.58; N = 252). Conversely, liraglutide demonstrated stronger obesity-associated signals for pancreatic cyst (ROR = 19.58, 95% CI = 2.03–188.45; N = 3), pancreatitis (ROR = 2.90, 95% CI = 1.91–4.39; N = 33), and biliary colic (ROR = 2.81, 95% CI = 1.43–5.55; N = 12).

### GLP-1RAs versus comparator drugs

In pooled analyses, GLP-1RAs demonstrated disproportionate reporting of gastrointestinal dysmotility and obstructive gastrointestinal AEs relative to non-GLP-1RA comparator drugs, including impaired gastric emptying (ROR = 41.83, 95% CI = 28.12–62.22; N = 1,136), intestinal obstruction (ROR = 40.51, 95% CI = 28.12–62.22; N = 417), constipation (ROR = 13.76, 95% CI = 11.23–16.86; N = 1,480), and diarrhea (ROR = 1.40, 95% CI = 1.30–1.52; N = 1,787). Additional AEs included suicidal ideation (ROR = 6.28, 95% CI = 3.83–10.28; N = 132) and optic ischemic neuropathy (ROR = 11.46, 95% CI = 6.21–21.16; N = 147). These pooled findings were directionally consistent with the primary comparative analyses.

## Discussion

This study is the first disproportionality analysis using the FAERS database to compare semaglutide and liraglutide across all adverse events while stratifying by therapeutic indication. We found (1) a consistent gastrointestinal safety signal reporting across GLP-1RAs, with evidence of heterogeneity between semaglutide and liraglutide and (2) notable ophthalmic and psychiatric AEs, again with heterogeneity between the two drugs. In addition to directly comparing semaglutide and liraglutide, we evaluated both medications against indication-specific comparators: metformin for type 2 diabetes and orlistat for obesity. Most prior analyses have evaluated GLP-1RAs as a therapeutic class irrespective of indication (Liu et al., 2022; Zhou et al., 2022; Osei et al., 2024), and most have focused on specific SOCs and AEs rather than comprehensively evaluating AE reporting patterns (Lu et al., 2025). Our findings provide some understanding of AE reporting patterns associated with GLP-1RAs and facilitate comparison of AEs across indications. These findings are not definitive due to the many limitations of FAERS but rather hypothesis-generating.

Gastrointestinal AEs represented the most consistent safety signals across analyses for GLP-1RAs. Given that GLP-1RAs substantially impact gastrointestinal motility and appetite regulation, we expected this finding, and it adds internal validity to the analysis (Shetty et al., 2022; Aldhaleei et al., 2024; Hepşen et al., 2025; Hepşen et al., 2026). More surprisingly, the magnitude of gastrointestinal AEs reporting differed.

Semaglutide demonstrated stronger disproportionality signals than liraglutide for gastrointestinal dysmotility and obstructive gastrointestinal AEs, including impaired gastric emptying and ileus, across both type 2 diabetes and obesity cohorts. Conversely, liraglutide demonstrated stronger disproportionality signals than semaglutide for cardiac and hepatobiliary disorders, including myocardial infarction and cholangitis, as well as neoplasm-related events such as metastatic breast cancer and uterine leiomyoma.

Patients prescribed semaglutide administer the drug once weekly, whereas patients prescribed liraglutide administer the drug daily. The extended half-life of semaglutide allows for sustained plasma exposure and more stable drug concentrations over time, potentially resulting in prolonged GLP-1 receptor stimulation (Yang and Yang, 2024). Such prolonged GLP-1 receptor stimulation slows gastric emptying, which may contribute to the higher frequency of gastrointestinal AEs noted in semaglutide reports.

In contrast, liraglutide demonstrated stronger signals for pancreatitis and hepatobiliary disorders, suggesting that heterogeneity may exist within the GLP-1RAs class despite shared mechanisms of action or that those administering liraglutide have unique medical characteristics that serve as confounders. These findings should be interpreted cautiously because spontaneous reporting systems cannot establish causality, and the prior literature has reported reductions in several cardiovascular and cancer-related outcomes with GLP-1RA use (Marso et al., 2016; Mann et al., 2017; McDonald et al., 2026).

The consistency of gastrointestinal safety signals across semaglutide, liraglutide, metformin, and orlistat supports the biologic plausibility of slowed gastrointestinal motility for GLP-1RAs. Importantly, the pooled GLP-1RAs analyses demonstrated markedly elevated RORs within many motility-related categories—impaired gastric emptying, ileus, intestinal obstruction, diarrhea, and constipation—relative to comparator medications, reaffirming the idea that gastrointestinal dysmotility is a class effect of GLP-1RAs.

We also identified notable ophthalmic and psychiatric AEs associated with GLP-1RAs. Ophthalmic AEs, including optic ischemic neuropathy and visual impairment, are established complications of type 2 diabetes, and we observed them more frequently among diabetes-indicated reports in our analysis. Studies have reported long-term vision benefits among GLP-1RA users, including a reduced risk of glaucoma and age-related macular degeneration, but rapid glycemic correction may temporarily exacerbate pre-existing microvascular disease in susceptible individuals (Huang et al., 2026). Researchers have observed this phenomenon in diabetic retinopathy, in which patients receiving GLP-1RAs may initially experience worsening of the condition before long-term benefits become apparent. The ophthalmic reports that we identified in FAERS are more likely to reflect underlying ophthalmologic vascular disease than a direct drug effect.

Psychiatric events, including suicidal ideation, appeared more frequently among type 2 diabetes-indicated reports. In 2023, the European Medicines Agency (EMA) noted elevated suicidal ideation reports related to GLP-1RAs, prompting investigation (EMA, 2023). Both the EMA and the FDA, in a similar evaluation, found no probable causal association between GLP-1RAs and suicidal ideation; however, the signal persists in FAERS, likely reflecting heightened reporting and the elevated baseline psychiatric burden associated with obesity (Ebrahimi et al., 2025; FDA, 2026).

A primary takeaway from our study is the consistency of gastrointestinal AEs. In a U.S. claims-based cohort from 2006 to 2020, patients receiving obesity-indicated semaglutide or liraglutide had higher risks of pancreatitis (aHR = 9.09, 95% CI = 1.25–66.00), bowel obstruction (aHR = 4.22, 95% CI = 1.02–17.40), and gastroparesis (aHR = 3.67, 95% CI = 1.15–11.90) than those prescribed bupropion–naltrexone (Sodhi et al., 2023). Although the magnitude of these effect estimates has been questioned (Suissa et al., 2024), our analysis identified similar gastrointestinal safety signals, with pancreatitis more frequently reported among obesity-indicated liraglutide reports than among semaglutide reports, and bowel-related adverse events more frequently reported among diabetes-indicated GLP-1RA reports than among metformin reports. In a cross-sectional analysis of 10,328 adults with type 2 diabetes and/or obesity from the National Institutes of Health All of Us cohort, liraglutide was associated with higher odds of abdominal pain (aOR = 1.12, 95% CI = 1.01–1.24), nausea and vomiting (aOR = 1.19, 95% CI = 1.01–1.45), and gastroparesis (aOR = 1.51, 95% CI = 1.21–1.91) than semaglutide. Pancreatitis risk did not differ between medications (aOR = 1.21, 95% CI = 0.92–1.61) (Aldhaleei et al., 2024). These large-scale findings support a consistent gastrointestinal signal; however, it is worth reiterating that causality cannot be established from spontaneous reporting data.

Considering limitations inherent to spontaneous reporting systems is relevant to interpreting our findings. Disproportionality analyses cannot establish incidence or causality and are susceptible to confounding by indication. We often lack detailed clinical information describing the individual patient within each report and are hostage to the reporter-assigned category. Conditions that could reasonably be expected to prompt a new prescription for a GLP1-RA—for example, hepatic steatosis among those with obesity—may therefore be reported as AEs by the original FAERS reporter.

FAERS is also subject to incomplete report information, differential report quality, and underreporting (Sakaeda et al., 2013). FAERS public data are standardized using MedDRA terminology that provides SOCs and PTs. Many reported AEs (for example, hepatic failure vs. drug-induced liver injury or intestinal obstruction vs. ileus) are likely to represent overlapping clinical entities. Although these categories could be combined, we chose to retain the given language of the dataset to preserve MedDRA comparability rather than introduce subjectivity by attempting to collapse clinically similar AEs.

Public and regulatory attention surrounding GLP-1RAs may additionally stimulate AE reporting and introduce notoriety bias, particularly among patients (Neha et al., 2021). Misclassification of therapeutic indication is also possible because FAERS reports are not linked to clinical records and depend on reporter-entered information.

Finally, because FAERS underwent a system redesign during the study period, we restricted analyses to standardized quarterly data releases rather than the continuously updated reporting system currently available (FDA, 2025). However, we do not anticipate that this approach altered the overall signal patterns identified.

Despite these limitations, this study has several notable strengths. We applied a multi-algorithm disproportionality framework requiring fulfilment of up to four signal detection criteria, reducing the likelihood that identified associations resulted from random variation alone. Additionally, rather than evaluating GLP-1RAs as a homogeneous class, we stratified analyses by medication type and indication, allowing partial consideration of confounding by indication.

## Conclusion

Semaglutide and liraglutide showed consistent patterns for AEs related to gastrointestinal slowing. Semaglutide was associated with greater reporting of gastrointestinal AEs than liraglutide, whereas liraglutide was associated with greater reporting of pancreatitis and liver injury, and this pattern held true regardless of the drug indication. Notable adverse events including ischemic optic neuropathy and suicidal ideation also showed positive signals, although these signals were less robust and warrant cautious interpretation. Future studies may aim to evaluate GLP-1RAs for the AEs identified within this post-marketing surveillance data.

## Data Availability

The data analyzed in this study are publicly available from the U.S. Food and Drug Administration (FDA) Adverse Event Reporting System (FAERS) at https://fis.fda.gov/extensions/FPD-QDE-FAERS/FPD-QDE-FAERS.html.
