# Supplementary material for: Indication-specific adverse event profiles of semaglutide and liraglutide: a FAERS disproportionality analysis
Source: Front Pharmacol. 2026 Sep 29;17:1936967. doi: 10.3389/fphar.2026.1936967 (PMC13658941; doi:10.3389/fphar.2026.1936967)
Supplement: Supplementary file 1 [file Supplementaryfile1.docx]

**Supplemental Methods**

Designated Medical Dictionary for Regulatory Activities (MedDRA) Indication Terms and Clinically Relevant SOCs

| **Indication terms** | **Clinically relevant SOCs** |
| --- | --- |
| Type 2 Diabetes (10067585, 10012601, 10029505, 10012611, 10026948, 10053247, 10045242)  Obesity (10029883, 10029885, 10027966, 10018743, 10090593, 10090594, 10090595, 10088937, 10050300, 10090518, 10049116) | Gastrointestinal disorders  Metabolism and nutrition disorders  Hepatobiliary disorders  Renal and urinary disorders  Cardiac disorders  Vascular disorders  Eye disorders  Nervous system disorders  Psychiatric disorders  Skin and subcutaneous tissue disorders  Immune system disorders  Endocrine disorders  Neoplasms benign, malignant and unspecified (incl cysts and polyps) |

Disproportionality Analysis 2x2 Table

|  | # of suspect AEs | # of other AEs | Total |
| --- | --- | --- | --- |
| Suspect drug | a | b | a + b |
| Other drug | c | d | c + d |
| Total | a + c | b + d | a + b + c + d |

Disproportionality analysis algorithms

| ***Algorithms*** | ***Formulas*** | ***Thresholds*** |
| --- | --- | --- |
| ***ROR*** | $ROR=(a/c)/(b/d)$ | $a \geq3$ |
|  | $SE (lnROR)=\sqrt{\frac{1}{a}+\frac{1}{b}+\frac{1}{c}+\frac{1}{d}}$ | $95\% CI \left( lower limit \right)$  $> 1$ |
|  | $95\%CI=e^{\ln(ROR)\pm1.96se}$ |  |
| ***PRR*** | $PRR=\frac{a/(a+b)}{c/(c+d)}$ | $a \geq3$ |
|  | $SE (lnPRR)=\sqrt{\frac{1}{a}-\frac{1}{a+b}+\frac{1}{c}-\frac{1}{c+d}}$ | $PRR \geq2$ |
|  | $\chi2=\frac{\left[ \left( ad-bc \right)^{2} \right]\left( a+b+c+d \right)}{\left[ \left( a+b \right)\left( c+d \right)\left( a+c \right)\left( b+d \right) \right]}$ | $\chi^{2}\geq4$ |
| ***EBGM*** | $EBGM=\frac{a(a+b+c+d)}{(a+c)(a+b)}$ | $EBGM05 > 2$ |
|  | $SE (lnEBGM)=\sqrt{\frac{1}{a}+\frac{1}{b}+\frac{1}{c}+\frac{1}{d}}$ |  |
|  | $95\%CI=e^{\ln(EBGM)\pm1.96se}$ |  |
| ***BCPNN*** | $IC=log_{2}\frac{p (x,y)}{p (x)p (y)}=log_{2}\frac{a(a+b+c+d)}{(a+b)(a+c)}$ | $IC025 > 0$ |
|  | $E (IC)=log_{2}\frac{(a+\gamma11)(a+b+c+d+\alpha)(a+b+c+d+\beta)}{(a+b+c+d+\gamma)(a+b+\alpha1)(a+c+\beta1)}$ |  |
|  | $V (IC)=\frac{1}{{(\ln2)}^{2}}[\frac{(a+b+c+d)-a+\gamma-\gamma11}{(a+\gamma11)(1+a+b+c+d+\gamma)}+\frac{(a+b+c+d)-(a+b)+a-\alpha1}{(a+b+\alpha1)(1+a+b+c+d+\alpha)}+\frac{(a+b+c+d+\alpha)-(a+c)+\beta-\beta1}{(a+b+\beta1)(1+a+b+c+d+\beta)}]$ |  |
|  | $\gamma=\gamma11\frac{(a+b+c+d+\alpha)(a+b+c+d+\beta)}{(a+b+\alpha1)(a+c+\beta1)}$ |  |
|  | $IC-2SD=E (IC)-2\sqrt{V (IC)}$ |  |
